# The change in age distribution of CAP population in Korea with an estimation of clinical implications of increasing age threshold of current CURB65 and CRB65 scoring system

**DOI:** 10.1371/journal.pone.0219367

**Published:** 2019-08-15

**Authors:** Byunghyun Kim, Joonghee Kim, You Hwan Jo, Jae Hyuk Lee, Ji Eun Hwang

**Affiliations:** Department of Emergency Medicine, Seoul National University Bundang Hospital, Bundang-gu, Seongnam-si, Gyeonggi-do, Republic of Korea; University of Florence, ITALY

## Abstract

**Background:**

CURB65 and CRB65 score are simple and popular methods to estimate the mortality in patients with community-acquired pneumonia (CAP). Although there has been a global increase in life expectancy and population ageing, we are still using the same age threshold derived from patients in late 1990s to calculate the scores. We sought to assess the implication of using higher age threshold using Korean population data and a single center hospital records.

**Methods:**

Using Korean National Health Insurance Service-National Sample Cohort (NHIS-NSC), we analyzed annual age distribution of CAP patients in Korea from 2005 to 2013 and report how patients aged >65 years increased over time. We also assessed annual change in test characteristics of various age threshold in Korean CAP population. Using a single center hospital registry of CAP patients (2008–2017), we analyzed test characteristics of CURB65 and CRB65 scores with various age thresholds.

**Results:**

116,481 CAP cases were identified from NHIS-NSC dataset. The proportion of patients aged >65 increased by 1.01% (95% CI, 0.70%-1.33%, P<0.001) every year. In the sample Korean population dataset, age threshold showed its peak AUROC (0.829) at 70. In the hospital dataset, 7,197 cases were included for analysis. The AUROC of both CRB65 and CURB65 was maximized at 71. When CRB71 was applied instead of CRB65 for hospital referral using score <1 to define a low-risk case, the potential hospital referral was significantly decreased (72.9% to 64.6%, *P*<0.001) without any significant increase in 1-month mortality in the low risk group (0.6% to 0.7%, *P* = 0.690).

**Conclusion:**

There was a significant age shift in CAP population in Korea. Increasing the current age threshold of CURB65 (or CRB65) could be a viable option to reduce ever-increasing hospital referrals and admissions of CAP patients.

## Introduction

Community-acquired pneumonia (CAP) is a considerable cause of mortality and morbidity in developed countries [1,2]. The prognosis of CAP depends on age, sex, underlying morbidities and causative organism [3,4]. CURB65 (or CRB65) and the Pneumonia Severity Index (PSI) are widely used as scoring systems for predicting prognosis using patient factors [5,6]. CURB65 is simple to calculate and is recommended for assessing the severity of pneumonia in hospital settings [7]. On the other hand, CRB65 (a simplified version of CURB65 without blood urea component), is intended to be used in clinics to help clinicians to make decisions such as hospital referrals [7].

Various modifications of CURB65 and CRB65 have been reported to improve performance by adding other factors to the original versions [8–11]. Because one of the disadvantages of CURB65 is that its performance is reduced in older patients [12], an age-modified version, CURB-age, which adds one more point to patients older than 85 years, was proposed. However, it has not been widely adopted because of its added complexity and decreased sensitivity compared to CURB65 [8,9].

It has been many years since CURB65 was introduced in 2003. In addition, CURB65 itself was derived from 1998 and 1999 patient data [6]. From 2000 to 2015, the average life expectancy at birth increased from 66.4 to 71.4 years globally, and from 76.3 to 82.3 years in South Korea [13]. The elderly population, especially those aged 65 and over, is growing rapidly [14]. Given the extended life expectancy and increased elderly population, we thought an alternative age threshold higher than the current one might provide increased classification performance while maintaining similar safety profile.

The objectives of the study was twofold. The first one was to report the temporal change of age distribution in the CAP population and how performance characteristics of current age threshold (65 years old or more) have changed in Korean population. The second one was to identify an alternative age threshold for CURB65 and CRB65 scores and analyze how it would influence their predictive performances and safety profile.

## Methods

This was a retrospective observational study using a population dataset and a hospital registry. The population dataset was the National Health Insurance Service-National Sample Cohort (NHIS-NSC, 2002–2013), a sample cohort representing whole Korean population. The NHIS-NSC cohort is a population-based cohort with de-identified claim information of 1 million individuals who were randomly sampled from the entire Korean population using stratification [15]. It provides diagnostic codes based on the International Classification of Diseases (ICD)-10 coding system, prescription and procedure codes and related costs, as well as demographic information such as age as 5-year intervals, sex and household income level from 2002 to 2013. It also has information regarding disability and death based on national disability registration data and death certificates [15].

The hospital registry is the Seoul National University Bundang Hospital-Emergency department pneumonia (SNUBH-EDP) registry. The SNUBH-EDP registry is an ED-based registry of pneumonia beginning in April 2008 for patients treated in the study facility. The study facility is a 1,360-bed academic hospital with the annual ED census was over 90,000 in 2017. The registry provides demographic information, comorbidities, initial vital signs, laboratory results and 30-day mortality. The detailed descriptions of the cohort data can be found in previous papers [16–18].

### Case definition of NHIS-NSC cohort

The inclusion criterion was clinically treated pneumonia among adults aged 20 or older from 2005 to 2013. The exclusion criterion was hospital-acquired pneumonia defined by a pneumonia event with any hospital stay (inpatient setting) within 10 days preceding the event. A pneumonia case was defined by temporally clustered outpatient or inpatient claims with a primary diagnosis of pneumonia (J10.0x, J11.0x, J12.x, J13.x, J14.x, J15.x, J16.x, J17.x, and J18.x) with any prescription for systemic antibiotics (Anatomical Therapeutic Chemical classification system code J01x). We clustered outpatient and inpatient claims based on the duration of antibiotic prescription for the former and length of stay for the latter. If a claim followed the endpoint of its predecessor within a week, they were considered as the same episode. The primary outcome event was one-month mortality. Because the NHIS-NSC cohort provides only the month and year of death instead of the actual date of death, we used death in the same or following month as a substitute for 1-month mortality. Comorbidities including diabetes mellitus, hypertension, ischemic heart disease, stroke, heart failure, chronic renal failure, advanced liver disease, chronic obstructive pulmonary disorder and malignancy were also assessed based on the claims of the two-year period preceding the CAP event. The detailed criteria used to define the covariates are displayed in S1 Table.

### Case definition from SNUBH-EDP registry

We included community-acquired pneumonia visits to the ED between April 2008 and March 2017. CAP patients aged 18 or older were included. Patients were excluded if they had one or more of the following: (1) Hospital-acquired pneumonia (HAP) and/or ventilator-associated pneumonia (VAP): (2) Patients with tuberculosis, HIV infection, or obstructive pneumonia due to cancer: and (3) Transfer to another hospital from the ED.

### Statistical analysis

Categorical variables were reported using frequencies or proportions, whereas continuous variables were reported using the mean or median value with standard deviation. Student’s t-test, Wilcoxon’s rank-sum test, Chi-squared test, or Fisher’s exact test were performed, as appropriate, for comparisons between the groups.

Using the population dataset (NHIS-NSC), we first assessed the predictive performance of different age thresholds increasing from age 50 to 80 in 5-year increments, calculating the area under the receiver operating characteristic curve (AUROC), sensitivity, specificity, positive predictive value (PPV) and negative predictive value (NPV). We compared their prognostic performances to that of current age threshold (65 years). AUROCs were compared using DeLong’s test. Sensitivity and specificity were compared using McNemar’s test. PPV and NPV were compared using relative predictive values [19]. We assessed annual trends of the change of sensitivity, specificity, PPV and NPV of the current age threshold and the alternative age threshold.

Using the hospital registry (SNUBH-EDP registry), we assessed the predictive performance of the clinical scores (CURB and CRB) using different age thresholds increasing from age 65 to 75 in 1-year increments, calculating the AUROC, sensitivity, specificity, PPV and NPV to identify a new age-threshold that maximized the AUROC of the scores. We calculated the number of patients expected to be discharged based on the low-risk criteria (CRB score = 0 and CURB score<2) when the current and the alternative age criteria were applied. We also compared the risk of having false negative result for 30-day mortality when the two different age thresholds were applied.

All data were presented with 95% confidence intervals (CIs) and *P*-values < 0.05 were considered significant. All the data handling and statistical analyses were performed using R-packages, version 3.3.2 (R Foundation for Statistical Computing, Vienna, Austria) and STATA (version 13; StataCorp, College Station, TX).

### Ethics statement

The institutional review board of SNUBH approved the analysis and waived the requirement for informed consent (approval No. B-1808-484-107).

## Results

### Baseline characteristics of study populations

Table 1 shows the baseline characteristics of subjects from the NHIS-NSC and SNUBH-EDP registry. Among 116481 subjects from the NHIS-NSC, 48385 (41.5%) were male and 33672 (28.9%) subjects were aged >65. The number of subjects treated in the inpatient setting was 15873 (13.6%) and 1-month mortality was 1439 (1.2%).

**Table 1:** Baseline characteristics of the study populations.

	NHIS-NSC	SNUBH-EDP registry
	(N = 116481)	(N = 7197)
Age, median (IQR)	NA	72 (58–80)
Population Age > 65 (%)	33672 (28.9)	4735 (65.8)
Sex, male (%)	48385 (41.5)	4384 (60.9)
Hypertension (%)	32519 (27.9)	2906 (40.4)
Ischemic heart disease (%)	9875 (8.5)	1279 (17.7)
Stroke (%)	5825 (5.0)	1644 (22.8)
Diabetes mellitus (%)	10354 (8.9)	1645 (22.8)
Chronic kidney disease (%)	4869 (4.2)	581 (8.1)
Chronic liver disease (%)	1241 (1.1)	301 (4.2)
Chronic obstructive pulmonary disease (%)	9058 (7.8)	838 (11.6)
Malignancy (%)	5703 (4.9)	1309 (18.2)
Admission rate (%)	15873 (13.6)	4041 (56.1)
30-day mortality (%)	1439 (1.2)	626 (8.7)
CRB65 class (%)		
0	NA	1949 (27.1)
1 or 2	NA	4779 (66.4)
≥ 3	NA	469 (6.5)
CURB65 class (%)		
0 or 1	NA	3788 (53.3)^a^
2	NA	1899 (26.8)^a^
≥ 3	NA	1412 (19.9)^a^
PSI class (%)		
I or II	NA	2310 (32.5)^a^
III	NA	1310 (18.5)^a^
IV or V	NA	3479 (49.0)^a^

NHIS-NSC, National Health Insurance Service-National Sample Cohort; SNUBH-EDP, Seoul National University Bundang Hospital-Emergency department pneumonia.

^a^ 98 patients without any variables were not included in calculation.

Among 7197 subjects from SNUBH-EDP registry cohort, 4384 (60.9%) subjects were male and 4735 (65.8%) subjects were aged >65. A total 4041 cases (56.1%) were treated in the inpatient setting and the 30-day mortality was 626 (8.7%). The number of high-risk patients based on CRB65 and CURB65 criteria (CRB65 score≥3 and CURB65 score≥3) was 469 (6.5%) and 1412 (19.9%), respectively.

### Annual trend in the age distribution of the Korean CAP population and the performance characteristics of the current age threshold

**Fig 1:**
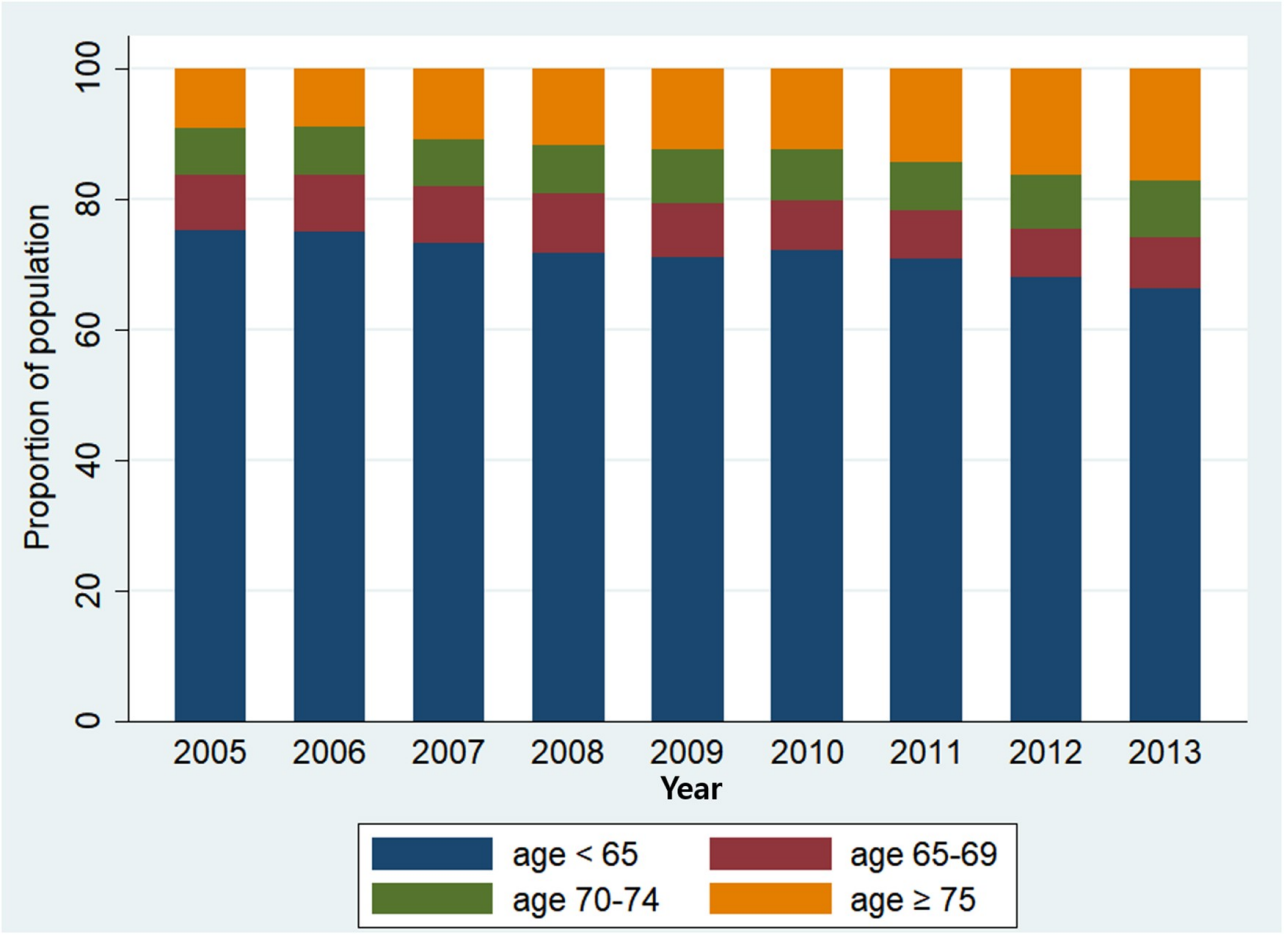
Annual age distribution of CAP patients in NHIS-NSC cohort.

**Fig 2:**
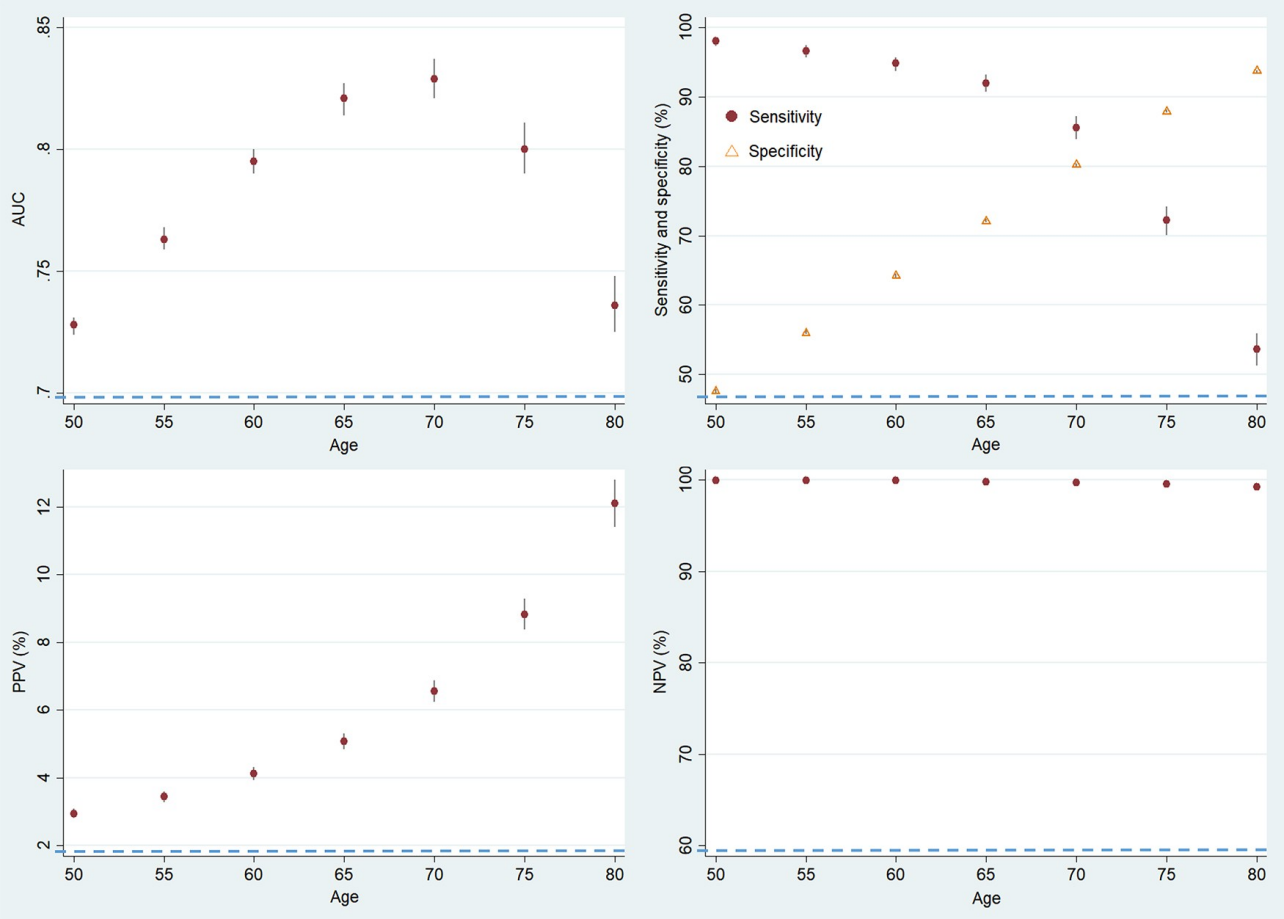
Sensitivity analysis of NHIS-NSC cohort with changing the age threshold. AUC, area under the receiver operating characteristic curve; PPV, positive predictive value; NPV, negative predictive value. The 95% confidence intervals for each point are shown as vertical lines.

Using the Korean population data (NHIS-NSC), we analysed the annual trend of change in age distribution of Korean CAP population and the performance characteristics of various age thresholds. Fig 1 shows the annual age distribution of CAP patients. The proportion of patients aged >65 increased every year (1.01%, 95% CI = 0.70 to 1.33%, P<0.001). Fig 2 shows the AUROC, sensitivity, specificity, PPV and NPV of the age threshold with increasing the threshold from 50 to 80 in 5-year increments. The AUROC was at maximum at 70 (0.829, 95% CI = 0.821 to 0.837) and the increase was statistically significant compared with 65 (0.821, 95% CI = 0.814 to 0.927, *P* = 0.005). When the cut-off age was increased from 65 to 70, sensitivity decreased from 92.0% to 85.6% (*P*<0.001), specificity increased from 72.1% to 80.2% (*P*<0.001), PPV increased from 5.1% to 6.6% (*P*<0.001) and NPV decreased from 99.8% to 99.7% (*P*<0.001) (S2 Table).

**Fig 3:**
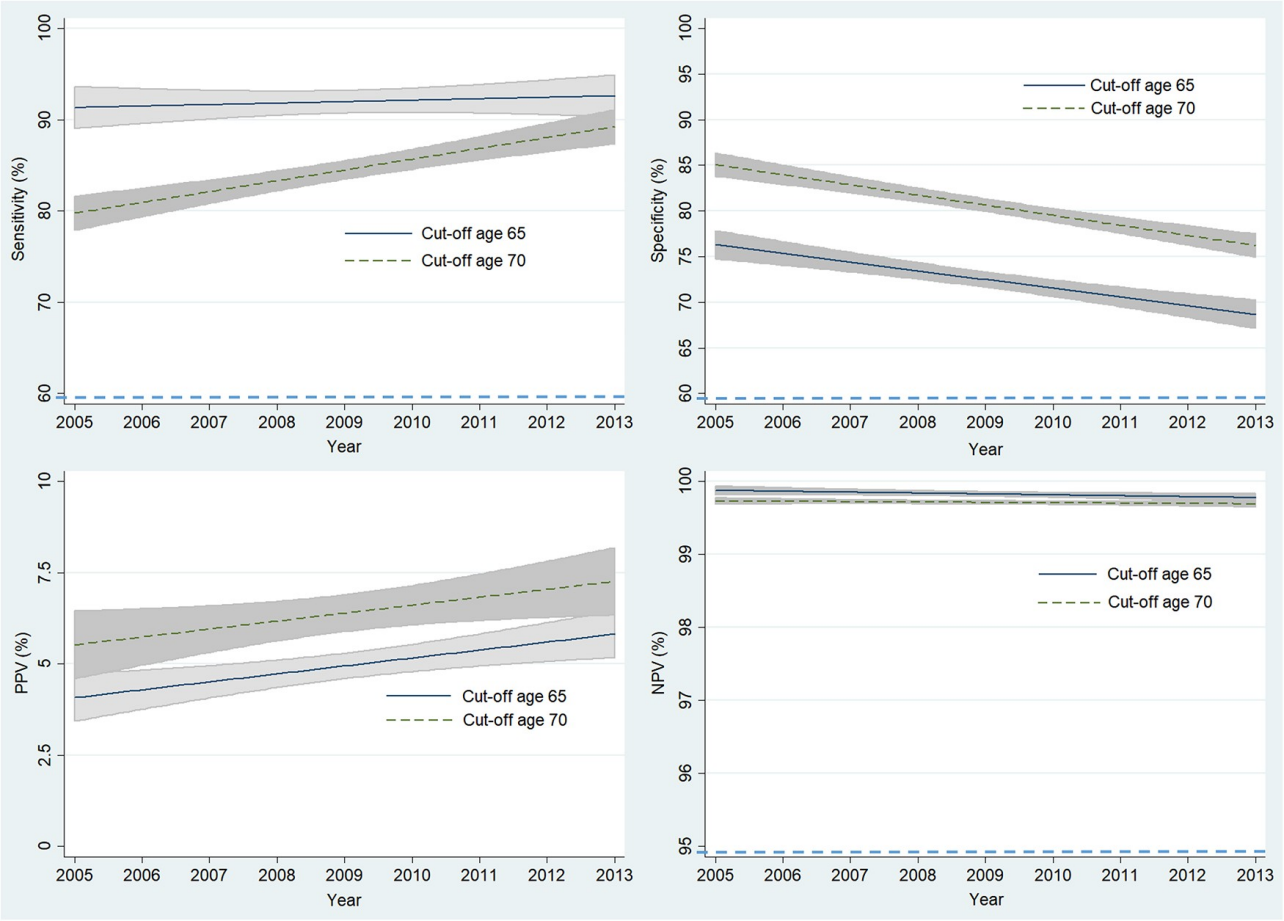
Annual trend in sensitivity, specificity, PPV and NPV of the current and alternative age thresholds in NHIS-NSC cohort. PPV, positive predictive value; NPV, negative predictive value. The 95% confidence intervals for each point are shown as shaded areas.

Fig 3 shows the annual trend in sensitivity, specificity, PPV and NPV of the current and alternative age thresholds. The sensitivity of the 65-year threshold did not change significantly; however, the sensitivity based on an alternative threshold (age 70) increased significantly, approaching the sensitivity of the 65-year threshold. The decreases in specificity were both significant with -1.0% (95% CI = -1.3% to -0.6%, *P*<0.001) and -1.1% (95% CI = -1.4% to -0.8%, *P*<0.001) annual decreases. PPV increased in both age thresholds with a 0.2% (95% CI = 0.1% to 0.4%, *P* = 0.007) annual increase at 65-years and a 0.2% (95% CI = 0.0% to 0.4%, *P* = 0.033) annual increase at 70-years. The decreases in NPV were small for both age thresholds, by -0.01% (95% CI = -0.02% to 0.00%) per year at 65-years and -0.01% (95% CI = -0.01 to 0.00%) per year at 70-years. The decreasing trend was statistically significant only at 65-years (*P* = 0.048).

### Identification of an alternative age threshold for CURB and CRB scores and an assessment of the performance change by the alternative age

Using the hospital registry data, we sought an alternative age threshold that would maximize the AUROC for both the CRB and CURB score systems. Table 2 shows the sensitivity, specificity, PPV, NPV, and AUROC for CRB and CURB with their age threshold increasing by one year. For both CRB and CURB, the AUROC was at maximum at 71, with AUROCs of 0.801 (95% CI = 0.785 to 0.817) and 0.828 (95% CI = 0.815 to 0.841), respectively.

**Table 2:** Performance changes of CRB65 and CURB65 with increasing age in SNUBH-EDP registry.

Score System (cut off score)	Sensitivity (%)	Specificity (%)	PPV (%)	NPV (%)	AUC	P-value^a^
**CRB-Age (≥1)**						
65	98.1	29.5	11.7	99.4	0.794	NA
66	98.1	30.7	11.9	99.4	0.796	.013
67	98.1	32.1	12.1	99.4	0.797	.010
68	97.7	33.6	12.3	99.4	0.797	.134
69	97.1	35.0	12.5	99.3	0.797	.199
70	97.1	36.6	12.7	99.3	0.799	.050
**71**	**97.0**	**38.5**	**13.1**	**99.3**	**0.801**	**.018**
72	95.7	40.4	13.3	99.0	0.798	.246
73	95.4	42.3	13.6	98.9	0.798	.278
74	93.9	44.2	13.8	98.7	0.794	.887
75	93.5	46.5	14.3	98.7	0.796	.577
**CURB-Age (≥2)**						
65	93.9	58.4	17.7	99.0	0.824	NA
66	93.8	58.8	17.8	99.0	0.825	.035
67	93.6	59.4	17.9	99.0	0.826	.079
68	93.1	60.0	18.2	98.9	0.826	.313
69	92.4	60.7	18.3	98.8	0.826	.418
70	92.3	61.5	18.6	98.8	0.827	.118
**71**	**91.9**	**62.5**	**18.9**	**98.7**	**0.828**	**.069**
72	89.6	63.5	18.9	98.5	0.826	.565
73	89.0	64.4	19.2	98.4	0.826	.501
74	87.1	65.7	19.5	98.1	0.824	.942
75	85.6	67.2	19.9	98.0	0.826	.526

SNUBH-EDP, Seoul National University Bundang Hospital-Emergency department pneumonia; PPV, positive predictive value; NPV, negative predictive value; AUC, area under the curve.

^a^ Compared with AUC of CRB65 or CURB65 respectively, using DeLong’s test.

Table 2 also shows the impact of changing the current age threshold (65) to the alternative age (71) on the performance indicators of 30-day mortality when the criteria for hospital referral (CRB score≥1 and CURB score≥2) were used for patient disposition. When the alternative age threshold was applied instead of the current one, the sensitivity of the CRB score (hospital referral) for 30-day mortality decreased from 98.1% to 97.0% (-1.1%, 95% CI = -1.9 to -0.3, *P* = 0.008), while the specificity increased from 29.5% to 38.5% (9.0%, 95% CI = 8.4 to 9.7, *P*<0.001). PPV increased from 11.7% to 13.1% (1.4%, 95% CI = 1.2 to 1.5, *P*<0.001) and NPV decreased from 99.4% to 99.3% but the difference was small and statistically not significant (0.1, 95% CI = -0.3 to 0.1, *P* = 0.246). Likewise, when the threshold age increased from 65 to 71, the sensitivity for the CURB score decreased from 93.9% to 91.9% (-2.0%, 95% CI = -3.2 to -1.0, *P*<0.001) while the specificity increased from 58.4% to 62.5% (4.1%, 95% CI = 3.6 to 4.6, *P*<0.001). PPV increased from 17.7% to 18.9% (1.2%, 95% CI = 1.0 to 1.5, *P*<0.001) and NPV decreased from 99.0% to 98.7% (-0.3, 95% CI = -0.4 to -0.1, *P* = 0.004).

Table 3 shows the number of additional false negative cases (death within 30 days despite being classified as low risk) from using the new age threshold. For the CRB score, the number of patients classified as a low risk group (i.e. CRB score< 1) increased significantly from 1949 (27.1%) to 2551 (35.4%, *P*<0.001). Similarly, for the CURB score, the number of patients classified as a low risk group (i.e. CURB score< 2) increased from 3788 (53.4%) to 4068 (57.3%, *P*<0.001). Despite the increase in the numbers of low risk group patients, the increase in 30-day mortality from 12/1949 (0.6%) to 19/2551 (0.7%, *P* = 0.690) was not statistically significant in patients triaged by CRB criteria. Similarly, the increase in 30-day mortality was not significant (38/3788 (1.0%) to 51/4068 (1.2%), *P* = 0.294) in patients triaged as low-risk by CURB criteria.

**Table 3:** Relationship between increasing the cut-off age and risk of mortality of patients from SNUBH-EDP registry.

Score System (cut off score)	Mortality (%)	Sensitivity	Specificity	PPV	NPV
**CRB65**					
0	12/1949 (0.6)	100	0	8.7	NC
1	154/3160 (4.9)	98.1	29.5	11.7	99.4
2	288/1619 (17.8)	73.5	75.2	22.0	96.8
3	135/404 (33.4)	27.5	95.5	36.7	93.3
4	37/65 (56.9)	5.9	99.6	56.9	91.3
**CRB71**					
0	19/2551 (0.7)	100	0	8.7	NC
1	174/2805 (6.2)	97.0	38.5	13.1	99.3
2	269/1413 (19.0)	69.2	78.5	23.5	96.4
3	129/366 (35.2)	26.2	96.0	38.3	91.7
4	35/62 (56.5)	5.6	99.6	56.5	91.3
**CURB65**					
0	3/1662 (0.2)	100	0	8.8	NC
1	35/2126 (1.7)	99.5	25.6	11.4	99.8
2	224/1889 (11.8)	93.9	57.9	17.7	99.0
3	211/1054 (20.2)	58.0	83.8	25.6	95.4
4	117/302 (38.7)	24.2	96.8	42.2	93.0
5	34/56 (60.7)	5.5	99.7	60.7	91.2
**CURB71**					
0	4/2100 (0.2)	100	0	8.8	NC
1	47/1968 (2.4)	99.4	32.4	12.4	99.8
2	236/1761 (13.4)	91.8	62.0	18.9	98.8
3	189/930 (20.3)	54.0	85.6	26.5	93.0
4	116/287 (40.4)	23.7	97.0	43.5	91.6
5	32/53 (60.4)	5.1	99.7	60.4	91.2

SNUBH-EDP, Seoul National University Bundang Hospital-Emergency department pneumonia; PPV, positive predictive value; NPV, negative predictive value.

## Discussion

In this study, we observed changing age distribution of Korean CAP population using a nationally representative dataset. We also observed a significant decrease in specificity of current age threshold in prediction of 1-month mortality. We tested the predictive performance of an alternative age threshold (70) in Korean CAP population, which was associated with increase in PPV with a negligible decrease in NPV. Based on this finding, we sought an alternative age threshold that would maximize the predictive performance of both the CURB and CRB scores using a hospital registry. The overall predictive performance measured by the AUROC was at maximum at 71, and changing to this alternative age threshold did not have a significant detrimental effect on the safety profiles of either the CURB or CRB scores while significantly increasing the number of candidates for discharge to home in CAP patients visiting the ED. These suggest increasing the age threshold for both CURB and CRB score could be a reasonable option that would help to reduce unnecessary referral and/or admissions [20].

Rapid ageing population, mainly due to increased longevity and decreased child births, is becoming an important issue, especially in developed countries [14]. According to the United Nations (UN), the term “ageing society” is defined as a society in which the proportion of the population over 65 years exceeds 7%, and the term “aged society” is defined as a society in which the proportion of the population over 65 years exceeds 14% [14]. The rate of societal ageing varies from country to country. Most developed countries such as the United Kingdom, Germany, and France entered the “ageing society” category in the 1970s, while South Korea experiencing rapid ageing entered the “ageing society” category in 2000. And it has only been 17 years that South Korea to move to “aged society” in 2017 [21]. In 2030, South Korea is expected to enter the "super-aged society" where the population over 65 is more than 20%. The United States and United Kingdom are expected to reach the "super-aged society" at the similar time [22].

With this shift in population characteristics, the average age of CAP patients and their overall mortality rate has changed accordingly (S1 Fig) [23,24]. In addition, there could be other changes in the characteristics of patients and healthcare deliveries that will modifies overall mortality. However, we still have been using the same age criterion developed decades ago. Although guidelines recommended combining clinical judgement with the scoring systems for severity assessment, we believe scoring systems like CURB65/CRB65 still has significant impact on hospital referral and admission as well as other related clinical decisions. Therefore, it is noteworthy that the increase in the threshold from 65 to 71 years significantly increased the number of patients in the low-risk group, especially in CRB71 without large change in the safety profile of the scores. If the age threshold remains fixed at 65 years, the number of unnecessary hospital referrals and hospitalizations may increase with the rapid shift in population characteristics of CAP patients. We believe this could result in increased healthcare costs and other nosocomial infections [20].

It should be mentioned that mortality rates in the low risk group can increase if we increase the age threshold. Even though the change was not statistically significant in this study, it could be significant if a larger dataset had been used. The problem of increased mortality in low-risk group could be minimized with clinical and/or technological advancements. There were studies to improve the CURB65 system using simple test such as pulse oximetry or urinary antigen test [10,18]. These additional tests can be performed easily at a local clinic as well as at a hospital.

This study has several limitations. First, test characteristics of age thresholds were calculated every five year interval as NHIS-NSC provides categorized age group instead of exact age. Second, because the NHIS-NSC database does not provide detailed clinical information such as vital signs, we could not calculate the CURB65 and CB65 scores using the population cohort. Third, the 30-day mortality rate in the dataset could be overestimated because the NHIS-NSC provide the month of death instead of its exact date. Fourth, the hospital registry was from a single tertiary hospital which could be not representative of general CAP population.

## Conclusions

There has been a significant age shift in CAP patient population due to ageing population. Increasing the current age threshold for CURB65 (or CRB65), which was derived using patient data of late 1990s, could be a viable option to reduce ever-increasing hospital referrals and admissions of CAP patients.

## Supporting information

S1 Fig Annual trend in crude mortality and age-standardized mortality in NHIS-NSC cohort. Age-standardized mortality was calculated by the direct method using the WHO standard population.
(TIF)

S1 Table Operational definitions for comorbidities. (DOCX)

S2 Table Sensitivity analysis with increasing age in NHIS-NSC cohort. AUC, area under the receiver operating characteristic curve; PPV, positive predictive value; NPV, negative predictive value.
(DOCX)
