# Supplementary figures and images for: The change in age distribution of CAP population in Korea with an estimation of clinical implications of increasing age threshold of current CURB65 and CRB65 scoring system
Source: PLoS One. 2019 Aug 15;14(8):e0219367. doi: 10.1371/journal.pone.0219367 (PMC6695142; doi:10.1371/journal.pone.0219367)

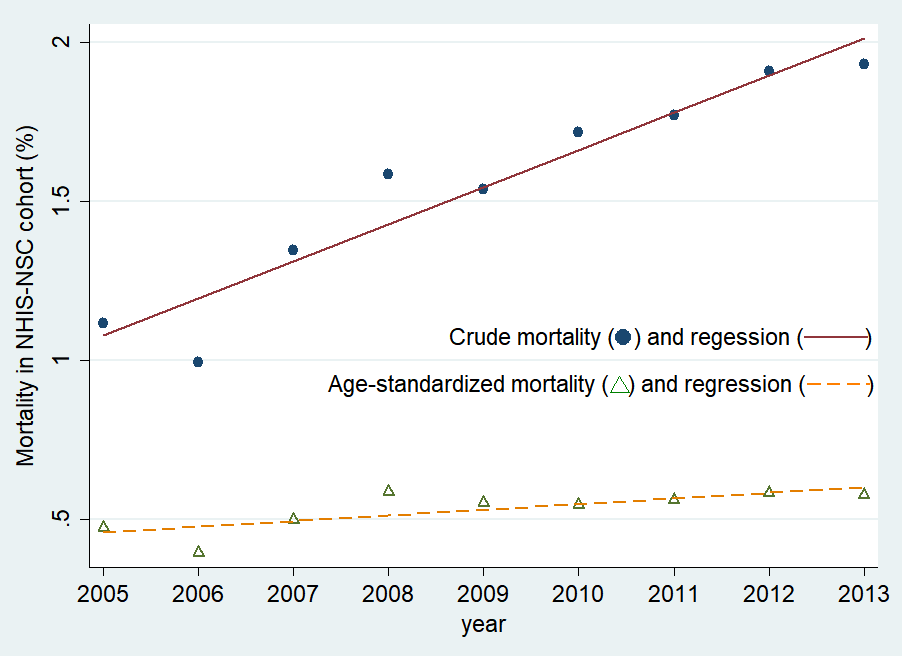

Supplement: S1 Fig — Age-standardized mortality was calculated by the direct method using the WHO standard population. (TIF) [file pone.0219367.s001.tif]
