# Supplementary material for: The change in age distribution of CAP population in Korea with an estimation of clinical implications of increasing age threshold of current CURB65 and CRB65 scoring system
Source: PLoS One. 2019 Aug 15;14(8):e0219367. doi: 10.1371/journal.pone.0219367 (PMC6695142; doi:10.1371/journal.pone.0219367)
Supplement: S1 Table — (DOCX) [file pone.0219367.s002.docx]

Coding conventions for the diagnostic codes

1. Dot (.) is omitted from diagnostic codes (i.e. J38.3 🡪 J383)
2. If there are more than a code in the same hierarchy (i.e. J38 and J383), the parent codes indicate only themselves specifically, but not its children (i.e. J38x).
3. The code at the end of each of hierarchy, including those without any parent, indicate both themselves and their children (i.e. J37x for J37)

**Supplementary table 1**. Operational definitions for comorbidities

|  | international Classification of Disease (ICD)-10 criteria | Scan range (past years) | | Minimum number of events | | Additional criteria |
| --- | --- | --- | --- | --- | --- | --- |
|  |  | Admission | Non-admission | Admission | Non-admission |  |
| Hypertension | I10, I11, I12, I13, I15 | 2 | 2 | 1 | 2 |  |
| Diabetes mellitus | E10, E11, E12, E13, E14 | 2 | 2 | 1 | 2 | Should be accompanied by oral hypoglycemic agent (ATC code: A10Bx) or insulin (A10Ax) |
| Chronic renal failure | E1021, E1121, E1221, E1321, E1421, E1022, E1122, E1222, E1322, E1422, I120, I131, I132, N18, N19 | 2 | 2 | 1 | 2 | Also include end-stage renal disease or any kidney disability registration |
| Ischemic heart disease | I20, I21, I22, I23, I24, I25 | 2 | 2 | 1 | 2 |  |
| Stroke | I60, I61, I62, I63,I64 | 2 | 2 | 1 | 2 |  |
| Chronic obstructive pulmonary disease | J43, J44 | 2 | 2 | 1 | 2 |  |
| Malignancy | C | 2 | 2 | 1 | 2 |  |

ATC: Anatomical Therapeutic Chemical Classification
