# Supplementary material for: The change in age distribution of CAP population in Korea with an estimation of clinical implications of increasing age threshold of current CURB65 and CRB65 scoring system
Source: PLoS One. 2019 Aug 15;14(8):e0219367. doi: 10.1371/journal.pone.0219367 (PMC6695142; doi:10.1371/journal.pone.0219367)
Supplement: S2 Table — AUC, area under the receiver operating characteristic curve; PPV, positive predictive value; NPV, negative predictive value. (DOCX) [file pone.0219367.s003.docx]

**Supplementary Table 2.** Sensitivity analysis with increasing age in NHIS-NSC cohort

| **Cut-off age** | **Sensitivity (%)** | **Specificity**  **(%)** | **PPV**  **(%)** | **NPV**  **(%)** | **AUC** |
| --- | --- | --- | --- | --- | --- |
| 50 | 98.0 | 47.6 | 2.9 | 99.9 | 0.728 |
| 55 | 96.6 | 56.1 | 3.4 | 99.9 | 0.763 |
| 60 | 94.8 | 64.2 | 4.1 | 99.9 | 0.795 |
| 65 | 92.0 | 72.1 | 5.1 | 99.8 | 0.821 |
| 70 | 85.6 | 80.2 | 6.6 | 99.7 | 0.829 |
| 75 | 72.2 | 87.9 | 8.8 | 99.5 | 0.804 |
| 80 | 53.6 | 93.7 | 12.1 | 99.2 | 0.736 |

PPV, positive predictive value; NPV, negative predictive value; AUC, area under the curve.
